# Comprehensive characterization of the prostate tumor microenvironment identifies CXCR4/CXCL12 crosstalk as a novel antiangiogenic therapeutic target in prostate cancer

**DOI:** 10.1186/s12943-022-01597-7

**Published:** 2022-06-18

**Authors:** Isabel Heidegger, Georgios Fotakis, Anne Offermann, Jermaine Goveia, Sophia Daum, Stefan Salcher, Asma Noureen, Hetty Timmer-Bosscha, Georg Schäfer, Annemiek Walenkamp, Sven Perner, Aleksandar Beatovic, Matthieu Moisse, Christina Plattner, Anne Krogsdam, Johannes Haybaeck, Sieghart Sopper, Stefanie Thaler, Markus A. Keller, Helmut Klocker, Zlatko Trajanoski, Dominik Wolf, Andreas Pircher

**Affiliations:** 1 Department of Urology, Medical University of Innsbruck, Innsbruck, Austria; 2 Medical University Innsbruck, Institute of Bioinformatics, Biocenter, Innsbruck, Austria; 3 University Hospital Schleswig Holstein, Institute of Pathology, Campus Lübeck, Lübeck, Germany; 4 Unicle Biomedical Data Science, Leuven, Belgium; 5 Department of Internal Medicine V, Medical University Innsbruck, Hematology and Oncology and Comprehensive Cancer Center Innsbruck (CCCI), Anichstreet 35, 6020 Innsbruck, Austria; 6 Department of Medical Oncology, University Medical Center Groningen, Groningen, The Netherlands; 7 Department of Pathology, Medical University Innsbruck, Innsbruck, Austria; 8 Medical University Innsbruck, Institute of Human Genetics, Innsbruck, Austria

**Keywords:** Prostate cancer, Tumor endothelial cell, Tip cell, Bulk RNA-seq, Single-cell RNA-seq, Target identification, CXCR4/CXCL12

## Abstract

**Background:**

Crosstalk between neoplastic and stromal cells fosters prostate cancer (PCa) progression and dissemination. Insight in cell-to-cell communication networks provides new therapeutic avenues to mold processes that contribute to PCa tumor microenvironment (TME) alterations. Here we performed a detailed characterization of PCa tumor endothelial cells (TEC) to delineate intercellular crosstalk between TEC and the PCa TME.

**Methods:**

TEC isolated from 67 fresh radical prostatectomy (RP) specimens underwent multi-omic ex vivo characterization as well as orthogonal validation of both TEC functions and key markers by immunohistochemistry (IHC) and immunofluorescence (IF). To identify cell–cell interaction targets in TEC, we performed single-cell RNA sequencing (scRNA-seq) in four PCa patients who underwent a RP to catalogue cellular TME composition. Targets were cross-validated using IHC, publicly available datasets, cell culture expriments as well as a PCa xenograft mouse model.

**Results:**

Compared to adjacent normal endothelial cells (NEC) bulk RNA-seq analysis revealed upregulation of genes associated with tumor vasculature, collagen modification and extracellular matrix remodeling in TEC. PTGIR, PLAC9, CXCL12 and VDR were identified as TEC markers and confirmed by IF and IHC in an independent patient cohort. By scRNA-seq we identified 27 cell (sub)types, including endothelial cells (EC) with arterial, venous and immature signatures, as well as angiogenic tip EC. A focused molecular analysis revealed that arterial TEC displayed highest CXCL12 mRNA expression levels when compared to all other TME cell (sub)populations and showed a negative prognostic role. Receptor-ligand interaction analysis predicted interactions between arterial TEC derived CXCL12 and its cognate receptor CXCR4 on angiogenic tip EC. CXCL12 was in vitro and in vivo validated as actionable TEC target by highlighting the vessel number- and density- reducing activity of the CXCR4-inhibitor AMD3100 in murine PCa as well as by inhibition of TEC proliferation and migration in vitro.

**Conclusions:**

Overall, our comprehensive analysis identified novel PCa TEC targets and highlights CXCR4/CXCL12 interaction as a potential novel target to interfere with tumor angiogenesis in PCa.

**Graphical Abstract:**

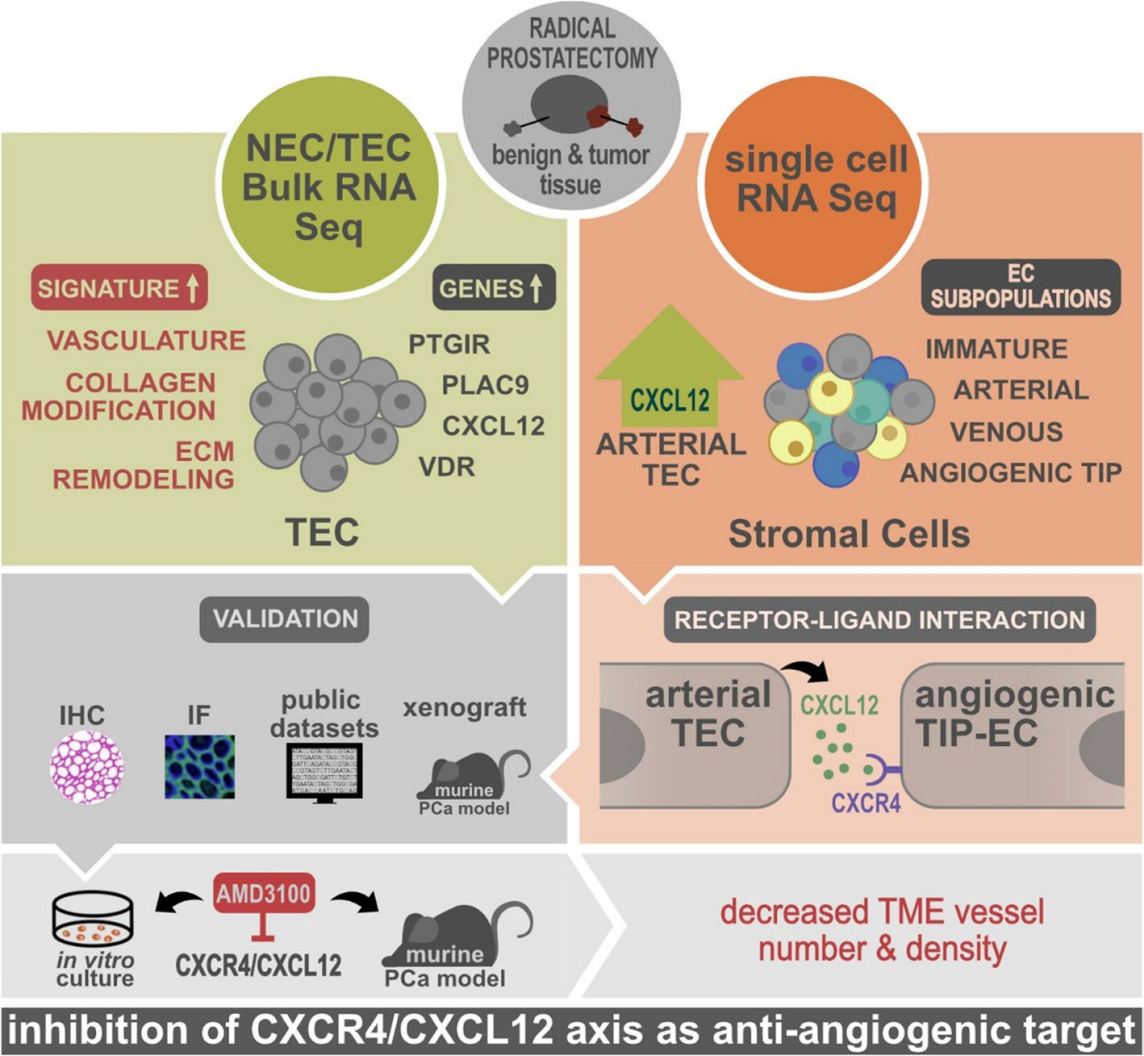

**Supplementary Information:**

The online version contains supplementary material available at 10.1186/s12943-022-01597-7.

## Background

The tumor microenvironment (TME) represents a highly dynamic and versatile network with inherent complexity during cancer progression and therapeutic modulation [1, 2].

Generally, the tumor stroma can exert suppressing and/or promoting effects in cancer. Thus, in the past years, the development of novel anti-cancer strategies focused on targets within the TME like immunotherapy or anti-angiogenic agents that entered successfully in clinical practice in multiple tumor entities [3, 4].

Prostate cancer (PCa) represents the most common malignancy in men and it is one of the leading causes of global cancer deaths [5]. The majority of prostate tumors are classified as adenocarcinomas since they originate from epithelial cells of the prostatic gland. In contrast to other malignancies, the mechanisms of interactions between stromal and epithelial cells are poorly defined. However, there is increasing evidence that stromal cells significantly contribute to the development of metastatic disease and androgen deprivation therapy resistance [6].

While endothelial cells (EC) under physiological conditions are well characterized in their functional properties, recently novel technologies like single cell RNA sequencing (scRNA-seq) helped to describe tumor endothelial cells (TEC) as novel cell type within the TME. Of note, TEC harbor an altered morphologic and genetic phenotype including structural chromosomal changes and mutations compared to normal endothelial cells (NEC) [7–10]. Lately, molecular analyses have identified differences between TEC and NEC in several cancer types, but so far, prostate TEC have not been characterized using advanced molecular profiling approaches [2, 9, 11].

Hence, we provide the first in depth characterization of PCa TEC using bulk RNA-seq, single-cell RNA-seq and functional assays to understand the molecular mechanisms and to identify potential targets within the complex PCa/stroma cell interaction.

## Materials and methods

Reagents and resources used in the study are listed in Table S1.

### EC isolation and characterization

The local ethics committee (EK no. 1017/2018; 1072/2018) approved the use of tissue samples obtained from fresh radical prostatectomy (RP) specimens. Written informed consent is available from all patients. Within a maximum of 1 h after surgical removal of the prostate, the specimen was inked and sliced. From a central slice 0.8 cm biopsies were punched from highly tumor suspicious areas (peripheral zone) as well as from a usually benign area (transitional zone) selected by an experienced uro-pathologist (GS) based on macroscopical morphology. To assure the tissue identity of paired biopsies the top of each biopsy (malign and benign area) was formalin-fixed, dehydrated and paraffin-embedded. Diagnostic eosin-hematoxylin (H&E) as well as basal cell marker p63 (1:4, Roche, 05,867,061,001) / tumor cell marker AMACR (1:400, DAKO, M361601-2) double-immunostaining was performed to confirm the malignancy or benignity of the tissue. In addition, in all carcinoma samples even the exact histological tumor type (acinar and cribriform) as well as the Gleason Score was determined (Workflow illustrated in Fig. S1).

For EC isolation biopsies were rinsed with PBS, minced into one mm^3^ pieces and transferred to 5 ml of digestion solution (DMEM high glucose (Sigma-Aldrich) supplemented with L-glutamine-penicillin–streptomycin (100 U/ml, Sigma-Aldrich), 20 µl DNase (75 U/ml, Sigma-Aldrich), 2 ml Collagenase V (2%, Sigma-Aldrich) and Dispase II (2.5 U/ml, Gibco)) for 45 min in a water bath at 37 °C. After stopping the digestion using 5 ml endothelial cell growth medium (ECM) supplemented with supplement kit for endothelial cell growth medium (PELOBiotech) plus 2% HyClone™ fetal bovine serum (GE Healthcare Life Sciences) and centrifugation at 400 g for 5 min, cells were re-suspended in ECM/18% FCS, seeded into 6-well plates coated with 1 ml 0.2% gelatine (Sigma-Aldrich) and incubated at 37 °C and a constant 5% CO_2_ atmosphere. After 24 h of cultivation, ECM was changed to 2% FCS content. Upon detecting the first EC colonies (7 to 10 days after seeding), EC were purified using anti-CD31-coated magnetic beats (Miltenyi Biotec) according to the manufacturer´s instructions. CD31^+^ cells were further cultivated in ECM upon passage 2 to 4 for bulk RNA-seq. FACS analyses were performed to ensure the presence of EC using anti-CD31 (BD Biosciences, 303,106), anti-CD45 (BD Biosciences, 560,367) anti-CD326 (Miltenyi Biotec, 130–113-263) and anti-CD90 (BioLegend, 328,114) antibodies (Cytomics FC 500, Beckman Coulter). In addition, immunofluorescence (IF) staining was performed as described in 2.

### Immunofluorescence (IF)

Cells were seeded for 2–3 days in gelatine-coated culture slides (Falcon) until 80% confluency, IF procedure was performed as previously described by our group [12]. The following primary antibodies were incubated overnight at 4 °C: anti-CD31 (1:20, Dako, M0823), anti-VE-Cadherin (1:100, Abcam, ab33168), anti-SDF1 (1:200, Abcam, ab9797) and anti-VDR (1:100, Abcam, ab3508) diluted in PBS/1% BSA. The next day, cells were stained with anti-IgG FITC (1:50, Dako) and anti-IgG Texas Red (1:500, Life Technologies) secondary antibodies diluted in PBS/1% BSA for 60 min at room temperature. Microscopy was performed using a phase-contrast inverted fluorescence microscope Zeiss Axio Imager Z2 microscope (Zeiss).

### Cell proliferation

2000 cells were seeded in five replicates into 96-well plates coated with 0.2% gelatine in either ECM without or with 2% FCS. Cell proliferation of NEC and TEC was determined by the ^3^H-thymidine incorporation assay as previously described by our group [12] or by EZ4U assay (Cell Proliferation and Cytotoxicity Assay, Biomedica) according to the manufacturer’s instructions.

### Scratch wound migration assay

Using a 200 µl tip, a scratch wound was applied on confluent EC monolayers in a six-well plate and photographed (T0) cultured in ECM medium, without additional FCS (for cell starvation). After 6 h of cultivation, cells were again photographed. Migration was measured with the ImageJ software expressed as the percentage of wound closure (reduction of gap area at T6 in % of gap area at T0) [13].

### Cell area/nucleus area/Junction length

The junctional length was calculated by measuring the length of all segments of continuous and discontinuous junctions on confluent NEC and TEC stained for VE-Cadherin using ImageJ. The sum of all segments was considered the total junctional length (100%), and the sum of all continuous segments was calculated as the percentage of total junctional length. Furthermore, we quantified the cell area and nucleus area with ImageJ for TEC and NEC (*n* = 3) and calculated the percentage of nucleus size per cell area.

### Quantitiative RT-PCR analysis

To quantify CXCL12 and CXCR4 mRNA levels, we designed “real-time” RT-PCR assays, using eEF1A as reference gene. Total RNA was prepared from 2 × 10^6^ cells using TRIzol reagent (Invitrogen) according to the manufacturer’s instructions. Quantitative RT-PCR was performed with the Luna Universal Probe One-Step RT-qPCR kit (New England Biolabs) using CXCL12 (forward: TCAGCCTGAGCTACAGATGC and reverse: CTTTAGCTTCGGGTCAATGC), CXCR4 (forward: ACTACACCGAGGAAATGGGCT and reverse: CCCACAATGCCAGTTAAGAAGA) and EEF1A1-specific oligonucleotides (forward CACACGGCTCACATTGCA and reverse: CACGAACAGCAAAGCGACC). CXCL12 and CXCR4 mRNA expression were normalized to EEF1A1.

### RNA isolation and sequencing

#### Bulk RNA-seq

Total RNA was isolated using TRIzol (Invitrogen) followed by final purification through the Qiagen RNeasy MinElute kit (Qiagen). The resulting RNA was submitted to QuantSeq 3’-mRNA library preparation (Lexogen) and sequenced using Ion Proton Hi-Q chemistry (Ion Torrent).

#### Single-cell RNA-seq (scRNA-seq)

The single-cell suspensions of FACS sorted freshly isolated cells were converted to barcoded scRNA-seq libraries using the Chromium Next GEM Single Cell 3' Kit v3.1 (10 × Genomics), aiming for 6000 cells per library. Single samples were always processed in parallel to avoid batch-dependent bias. Multiplexed libraries were sequenced with the Illumina Novaseq sequencer (Illumina), aiming 50,000 reads per targeted cell.

Both bulk and scRNA-seq was carried out at the NGS core facility of Medical University Innsbruck.

### RNA-seq analyses

#### Data pre-processing

Bulk RNA-seq reads were mapped to the human genome (build GRCh38) using the STAR (v2.5.3a) package, and the mapped reads were assigned to features using the HTseq-count (v0.12.3) package. The scRNA-seq reads were mapped to the human genome (build GRCh38) using the CellRanger software (10 × Genomics, v3.1). Data from the raw, unfiltered matrix was analyzed using the UniApp platform (Unicle Biomedical Data Science).

#### Quality control and data normalization

Regarding the bulk RNA-seq dataset, genes expressed at a level of at least one count per million reads in at 30% of samples were filtered and normalized using the EdgeR package (v3.35.0). The following quality control steps were applied to the scRNA-seq dataset: (i) genes expressed by < 3 cells were not considered; (ii) cells that had either fewer than 201 (low-quality cells) or over 8,000 expressed genes (possible doublets), or over 20% of unique molecular identifiers (UMIs) derived from the mitochondrial genome were removed. The data of the remaining cells were natural-log transformed using log1p and normalized using Seurat (v4.0.5).

#### In silico high-quality cell selection

After auto-scaling, the normalized data were first summarized by principal component analysis (PCA), and the first 20 PCAs were visualized using t-Distributed Stochastic Neighbor Embedding (t-SNE, Rtsne package, v0.15) with a perplexity value of 100 and a learning rate of 100. Graph-based clustering was performed to group cells according to their gene expression profiles as implemented in Seurat. Cell clusters were annotated based on canonical markers. Cells that could not be assigned unambiguously assigned to a biologically meaningful phenotype and so might represent low-quality cells or doublets were excluded from the analysis.

#### Feature selection and dimensionality reduction

After in silico selection of high-quality cells, we identified genes with high variability using the Seurat FindVariableGenes function with default parameters. The normalized data were auto-scaled, and principal component analysis was performed on variable genes, followed by t-SNE to construct a two-dimensional representation of the data. This representation was only used to visualize the data.

#### Cluster identification

To estimate the number of distinct cell types, we color-coded t-SNE plots for each of the detected genes using the brute force module of the UniApp (Unicle Biomedical Data Science) and identified clusters of cells with discriminating gene expression patterns in all datasets. To unbiasedly group cells, we performed PCA on highly variable genes and used graph-based clustering as implemented in the FindClusters function of the Seurat package [14]. Cluster results were visualized using t-SNE to verify that all visually identified clusters were captured and not under-partitioned. Over-partitioned clusters representing the same biological phenotype were merged into a single cluster.

#### Pair-wise differential analysis

Differential expression analysis between TEC and NEC samples from the bulk RNA-seq dataset was performed using the limma R package (v3.49.0) as described previously [15, 16]. The same method was used to compare individual clusters in the scRNA-seq dataset.

#### Marker gene analysis

We used a two-step approach to obtain ranked marker gene lists for each cluster. As a first criterion, marker genes for a given cluster should have the highest expression in that cluster compared to all other clusters and are therefore uniquely assigned to one cluster. Second, we ranked marker genes using a product-based meta-analysis [17]. Briefly, we performed a pair-wise differential analysis of all clusters against all other clusters separately and ranked the results of each pair-wise comparison by log2-fold change. The most upregulated genes received the lowest rank (top-ranking marker genes), and the most downregulated ones received the highest. For each cluster, we combined the rank numbers for all genes in all pair-wise comparisons by calculating their product to obtain a final list of ranked marker genes for each cluster.

#### Cluster quantification

The representation of clusters across patients, tumor type, and tumor and peritumoral tissue was quantified using the normalized gene expression values as returned by limma, using the patient as a covariate and a *p*-value cutoff of < 0.05 to call differences in fractional composition. Statistical hypothesis tests were performed using two-sample location t-tests where applicable.

#### Cluster annotation

We annotated clusters based on literature-curated marker genes of canonical marker genes. We used a three-step approach to identify a putative biological function in case of an entirely unknown phenotype or new sub-lineages of a canonical phenotype, which could not be annotated based on canonical marker genes or gene sets. First, we searched through the top 50 ranking list of markers for a coherent set of genes involved in similar biological processes. Second, if we identified a putative signature, we determined whether other genes associated with such a signature were also highest expressed in this phenotype. Third, we integrated insights from additional into our assessment.

#### Heatmap analysis

For bulk transcriptomics, gene expression heatmaps are based on averaged auto-scaled data. The heatmaps regarding the scRNA-seq dataset are based on cluster-averaged gene expression to account for cell-to-cell transcriptomic stochastics. Data was auto-scaled for visualization. All heatmaps were produced using the heatmaply R package.

#### Re-analysis of publicly available transcriptome datasets

We determined CXCL12 expression in previously published murine and TEC *versus* NEC transcriptomics datasets using data from a previously published meta-analysis [18]. The expression data were normalized using the counts per million (CPM) function available from the EdgeR package, and then log2 transformation was applied to the resulting CPM values.

#### TCGA analysis

Raw count gene expression data were obtained from the Prostate adenocarcinoma (PRAD) cohort catalogued in The Cancer Genome Atlas [19] and processed as in our recent publication followed by log 2 transformation [20].

We performed survival analyses on both the gene expression data, as well as on the pathway level. To calculate per-sample pathway enrichment scores, we performed Gene Set Variation Analysis (GSVA, v1.41.0). GSVA scores were only calculated for EC-enriched marker gene sets with a minimum of five detected genes, all other parameters were default [21]. Downstream analysis was performed on the output of GSVA to test gene expression signatures. The optimal cut-point used for the stratification of the PRAD cohort (“low” and “high” expression) was calculated using the maximally selected rank statistics from the maxstat' R package. The cut-off point for CXCL12 expression was calculated as a log2 expression value of 17,626. Survival analysis was performed via the Kaplan–Meier estimator using the log-rank test to determine significance. A univariate Cox proportional hazard model was fitted to the data to infer the hazard ratios (HR).

#### Gene set enrichment analysis

We used gene set enrichment analysis (GSEA) as implemented in the clusterProfiler package to compare gene expression signatures between groups [22]. Gene set analysis was performed using a set of vascular-related gene sets selected from the Molecular Signatures Database (MSigDB version 7.41 downloaded from http://bioinf.wehi.edu.au/software/MSigDB/), a collection of expert annotated gene sets. GSEA scores were calculated for sets with a minimum of 5 (scRNA-seq) or 10 (bulk RNA-seq) detected genes; all other parameters were default.

### Immunohistochemistry (IHC)

#### Patient tissue

The cohort included tissue from 152 primary PCa and 34 benign prostatic tissue samples of patients diagnosed with PCa between 1998 and 2014 in the Institute of Pathology, Hospital Goeppingen. IHC analysis of tissues was performed on tissue microarrays (TMA). Briefly, formalin-fixed paraffin-embedded tissues were cut in 4 µm sections, mounted on slides and relevant tissue regions were circled by a pathologist. Three representative cores of the circled regions measuring 0.6 mm in diameter from each sample were assembled into tissue microarray blocks using a semiautomatic tissue arrayer. IHC staining was performed using the Ventana Discovery System (Ventana Medical System). Briefly, slides were incubated at room temperature with the following primary antibodies: anti-CD31 (Sigma-Aldrich, 131M9), anti-PTGIR (Sigma-Aldrich, WH0005739M1), anti-PLAC9 (Novus Biologicals, NBP1-86,202), anti-CXCL12 (Thermo Fisher Scientific, PA5-95,549), anti-VDR (Sigma-Aldrich, WH0007421M2), and detected with the ultraView Universal DAB Detection Kit (Ventana Medical System). Evaluation of PTGIR-, PLAC9-, CXCL12- and VDR expression on EC in the prostatic fibromuscular tissue was independently performed visually by two pathologists (AO and SP). Staining for the EC marker CD31 and correlation with hematoxylin/eosin stain supported the identification of vessels. For IHC validation of identified tip and artery markers, we used FBLN5 (Sigma-Aldrich, HPA000848), INSRß (Cell Signalling, #23,413) and ENPP2 (Autotaxin) (Phoenix Pharmaceuticals, H-008–29) and androgen receptor (AR) Roche Tissue Diagnostic ­antibody SP107) antibodies in 5 independent PCa tissue samples.

### Vessel characterization in a mouse xenograft model

For the experimental study, we refer to the original publication [23]. Formalin-fixed and paraffin-embedded (FFPE) tissue slides from male Hsd:Athymic Nude-Foxn1nu 6 to 8 weeks old injected with 3 × 10^6^ PC3-Luc cells and treated by sterile water intraperitoneally (control) or AMD3100 3.5 mg/kg ip daily five times per week (treatment group) were investigated for CD31^+^ vessels. Therefore, we used the anti-mouse CD31 antibody (Cell Signaling, D8V9E). IHC was performed on the Ventana Discovery Ultra (Ventana Medical System).

## Results

### Isolation, cultivation and characterization of TEC and NEC from human prostate tissue

**Fig. 1:**
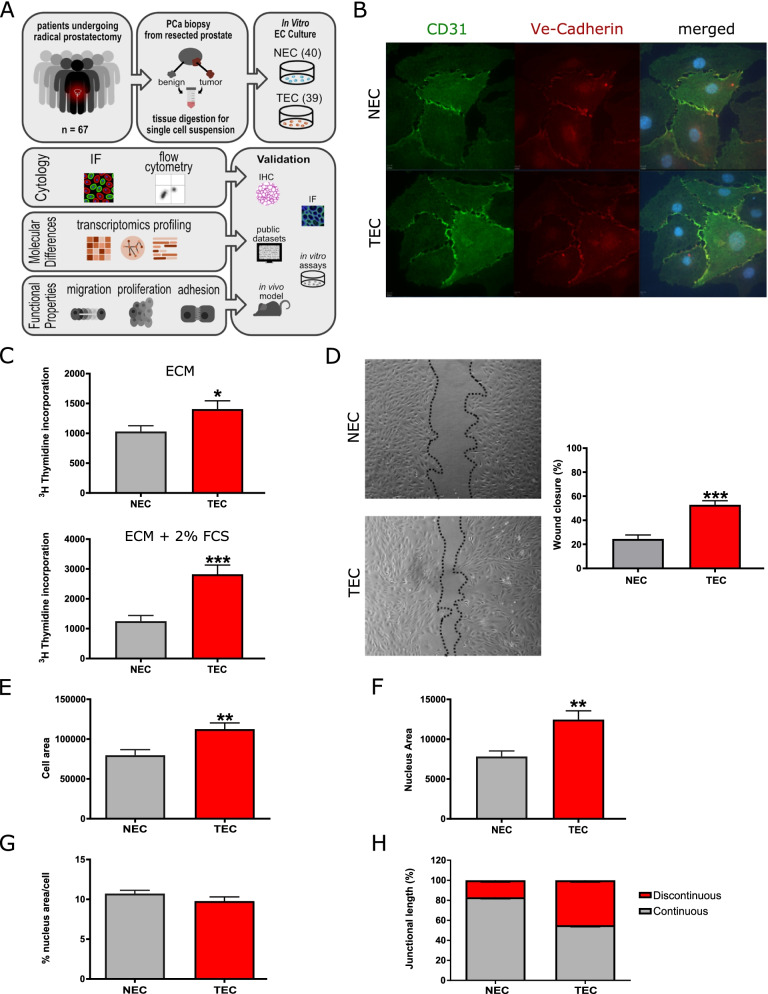
Functional characteristics of cultured TEC. **A** Study design. PCa, prostate cancer; NEC, normal endothelial cells; TEC, tumor endothelial cells. **B** Representative immunofluorescence staining (CD31 and VE-Cadherin) of NEC and TEC. **C** ^3^H-thymidine incorporation assay with NEC and TEC cultivated in ECM or ECM + 2% FCS (mean +—SEM; *n* = 4, *p* < 0.001). **D** Micrographs (left) and quantification (right) of NEC and TEC migration in scratch wound assay (mean +—SEM; *n* = 4, *p* < 0.001). **E** Quantification of cell area in TEC and NEC (mean +—SEM; *n* = 4, *p* < 0.01) **F** Quantification of nucleus area in TEC and NEC (mean +—SEM; *n* = 4, *p* < 0.01). **1G.** % nucleus area/cell in TEC and NEC (*n* = 4). **H** Quantification of continuous versus discontinuous junctions (junctional length, % of total junctional length) in TEC and NEC (*n* = 4)

NEC and TEC isolated from 67 fresh RP specimens of patients diagnosed with organ-confined PCa (Fig. 1A and Fig. S1 and S2) were purified and selected for the EC marker CD31. Patients’ characteristics are illustrated in Table S2. The endothelial phenotype of isolated PCa-derived TEC and NEC was confirmed by immunocytological detection of typical EC features (CD31/VE-Cadherin, representative image see Fig. 1B). The endothelial phenotype was further verified by FACS-based positive staining for CD31 and the absence of the leukocyte marker CD45 (data not shown). We cultured NEC and TEC until passage four in order to generate sufficient cell numbers and perform a comprehensive analysis on NEC and TEC including functional characteristics and bulk RNA-seq (Fig. 1A). Prostate TEC showed increased cell proliferation and migration when compared to NEC, (Fig. 1C-D), which is in line with previous reports for other cancer types [7, 11, 24]. In addition, prostate TEC exert morphological changes, such as an increased size of the overall cell (Fig. 1E) as well as the nucleus area compared to NEC (Fig. 1F), however, the nucleus to the cell ratio size was similar both EC types (Fig. 1G). In addition, more abundant discontinuous cell junctions were observed in TEC (Fig. 1H).

### RNA-Seq reveals differences between prostate TEC and NEC

**Fig. 2:**
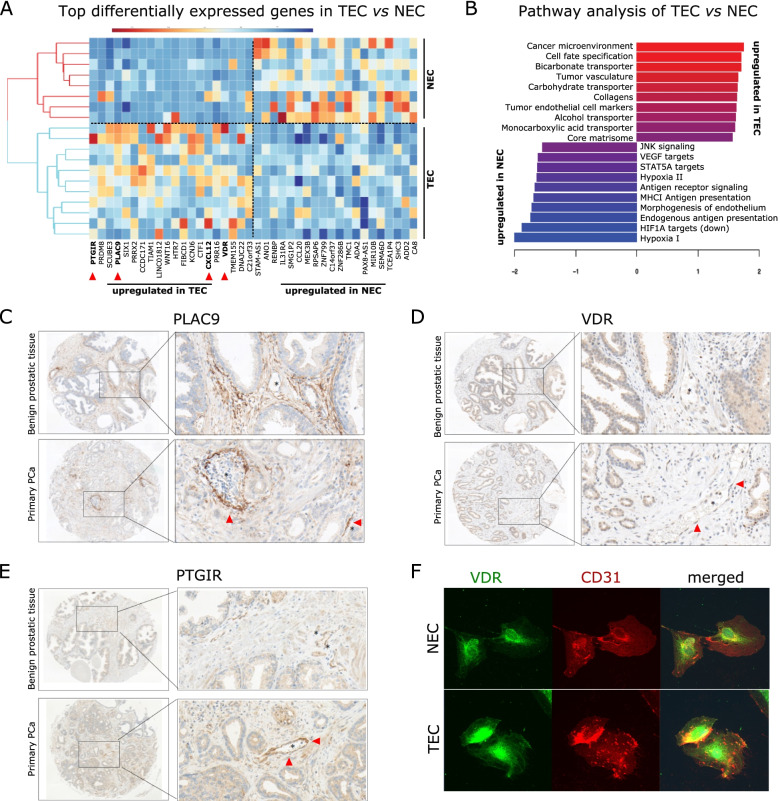
Bulk RNA-sequencing identified novel TEC markers. **A** Heatmap analysis and hierarchical clustering of the top 20 most up-and downregulated genes. Color-scale: red is higher expressed, blue is lower expressed. **B** Horizontal bar graphs representing the top 10 differentially expressed pathways as assessed by gene set enrichment analysis. **C** Micrographs of immunohistochemistry probing PLAC9. Note the high expression levels of PLAC9 in the tumor vasculature. **D** Micrographs of immunohistochemistry probing VDR. Note the high expression levels of VDR in the tumor vasculature. **E** Micrographs of immunohistochemistry probing PTGIR. Note the high expression levels of PTGIR in the tumor vasculature. **F** Representative immunofluorescence staining (CD31 and VDR) of NEC and TEC

We performed bulk RNA-seq of 5 paired TEC and NEC samples and 9 unpaired samples to identify molecular differences between prostate TEC and NEC. By differential gene expression analysis, we determined genes that drive differences between TEC and NEC and identified a 40-gene signature that separates TEC from NEC (Fig. 2A, Table S3). Among the most upregulated genes in this signature, we could descry PTGIR, PRDM8, PLAC9, CXCL12 and VDR, which depict interesting TEC targets and markers. The prostaglandin I2 receptor (PTGIR) was the most upregulated gene and has already been associated with mouse TEC in renal cell cancer [25]. Prostaglandin and prostacyclin signaling via the cognate receptor PTGIR regulates vasodilatation, inhibits platelet aggregation (anti-thrombotic) and modulates vascular smooth muscle cell proliferation. PR domain-containing 8 (PRDM8) was described to be expressed in hepatocellular TEC however the function in PCa EC has not been described yet [26].

The placenta associated protein 9 (PLAC9) has been investigated in fetal heart EC and is associated with extracellular matrix remodeling [27]. CXCL12 (also known as SDF1) is an important cytokine in the PCa stromal compartment and associated with tumor aggressiveness [28]. The impact of vitamine D receptor (VDR) on TEC is not reported yet, however VDR affects vascular dysfunction by interacting with blood pressure control [29].

Gene set enrichment analysis (GSEA), using a manually curated list of EC-related gene sets from the molecular signatures database (MSigDB), revealed upregulation of genes known to be associated with the tumor vasculature (Fig. 2B). Noteworthy, processes previously associated with TEC in vivo*,* such as extracellular matrix biosynthesis and carbohydrate transport, also ranked in the top enriched processes (Fig. 2B). Other canonical TEC pathways upregulated in cultivated PCa TEC were involved in lactate transmembrane transport, collagen formation and matrisome formation (Fig. 2B). Consistent with the phenotypic characteristics of PCa TEC (see above), pathways that regulate cell adhesion to substrate, cell–cell adhesion and cell spreading were also upregulated (Table S4). In contrast, pathways that induce EC proliferation, morphogenesis, hypoxia and VEGF signaling were surprisingly downregulated (Fig. 2B). For orthogonal validation of selected RNA-seq identified TEC markers, we used an independent patient cohort of primary PCa (Fig. 2C-E). In total, 152 primary PCa samples and 34 benign prostatic tissue samples were stained for CD31 marking the endothelial inner lining of vessels. Summarizing, our results reveal a higher expression of all markers in TEC when compared to NEC from benign prostate samples. The percentage of CXCL12-, PTGIR-, PLAC9- and VDR-positive vessels in primary tumors are 48.7%, 20.1%, 10.1% and 24.5%, respectively. In comparison, the percentage of CXCL12-, PTGIR, PLAC9- and VDR-positive vessels in benign samples are 8.5%, 8.9%, 3% and 8.7%, respectively (Fig. 2C-E).

In addition, in vitro IF validation in cultured TEC and NEC confirmed significantly increased expression and EC specificity of VDR (Fig. 2F). Altogether, our results indicate that cultured prostate TEC maintain several characteristics of in vivo TEC.

### CXCL12 is a prostate TEC marker

**Fig. 3:**
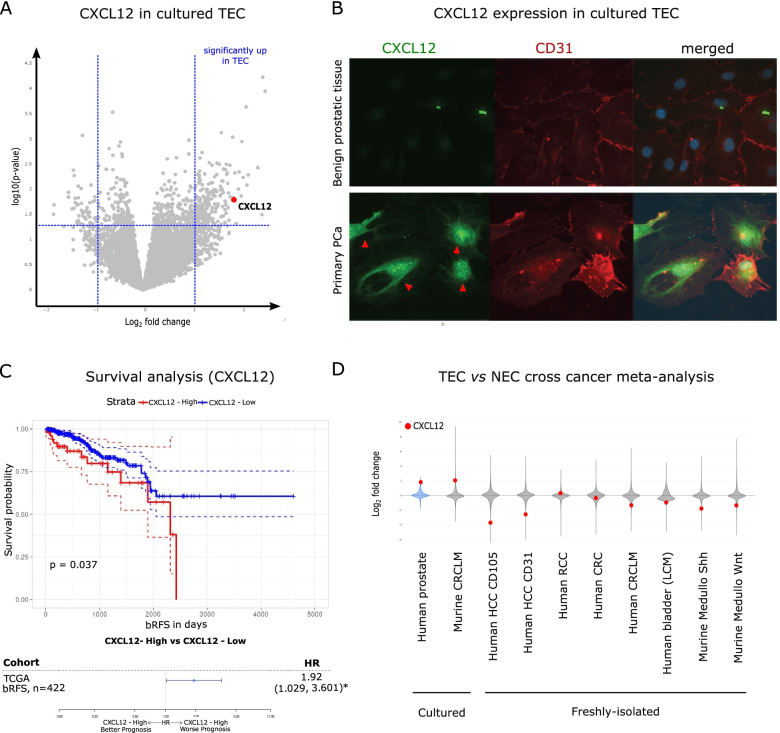
CXCL12 is a clinically relevant TEC marker. **A** Volcano plot representing a differential analysis of NEC versus TEC. **B** Representative immunofluorescence staining (CD31 and CXCL12) of NEC and TEC. Note the high expression levels of CXCL12 in the tumor vasculature. **C** Kaplan–Meier curves where patients are stratified based on the high or low expression of CXCL12. *n* = 422, source TCGA dataset. The CXCL12 expression cutoff was determined using the R package “maxstat”. The cut-off point for CXCL12 expression was calculated as a log2 expression value of 17.626. **D** Violin plots visualizing the log2 fold-change distribution in gene expression (gray area) in murine and human tumor EC versus their counterpart normal healthy EC. The red dot indicates where CXCL12 is located in the distribution. Data are based on a meta-analysis of publicly available transcriptome datasets

Differential analysis between cultured NEC and TEC revealed that CXCL12 was among the most upregulated genes, a finding that was confirmed using IF on cultivated EC (Fig. 3A-B and Table S3). Analysis of a publicly available data (TCGA PRAAD cohort) from a cohort of 422 PCa patients revealed that high CXCL12 expression is associated with a significantly higher rate of biochemical tumor recurrence (Fig. 3C). We further elaborated the role of CXCL12 interaction with its cognate receptor CXCR4 again using public available data and found that i) patients with aggressive cancers reflected by Gleason Score have higher CXCR4 expression levels, ii) the histological subtype of PCa does not influence CXCL12 or CXCR4 expression and iii) CXCR4 positively correlates with AR activity reflected by prostate specific antigen (PSA) expression (Fig. S3).

To determine whether CXCL12 is also altered in TEC in other tumor types, we performed a cross tumor meta-analysis [18, 30]. Specifically, we used publicly available TEC and NEC transcriptomics datasets and performed a differential analysis in each dataset separately. We then visualized the position of CXCL12 in the distribution of differentially regulated genes. Analysis of this independent data set showed that CXCL12 was downregulated in most other tumor entities but highly upregulated in cultured prostate TEC (Fig. 3D).

### Single-cell mapping of the prostate TME identifies CXCL12-producing cell types

**Fig. 4:**
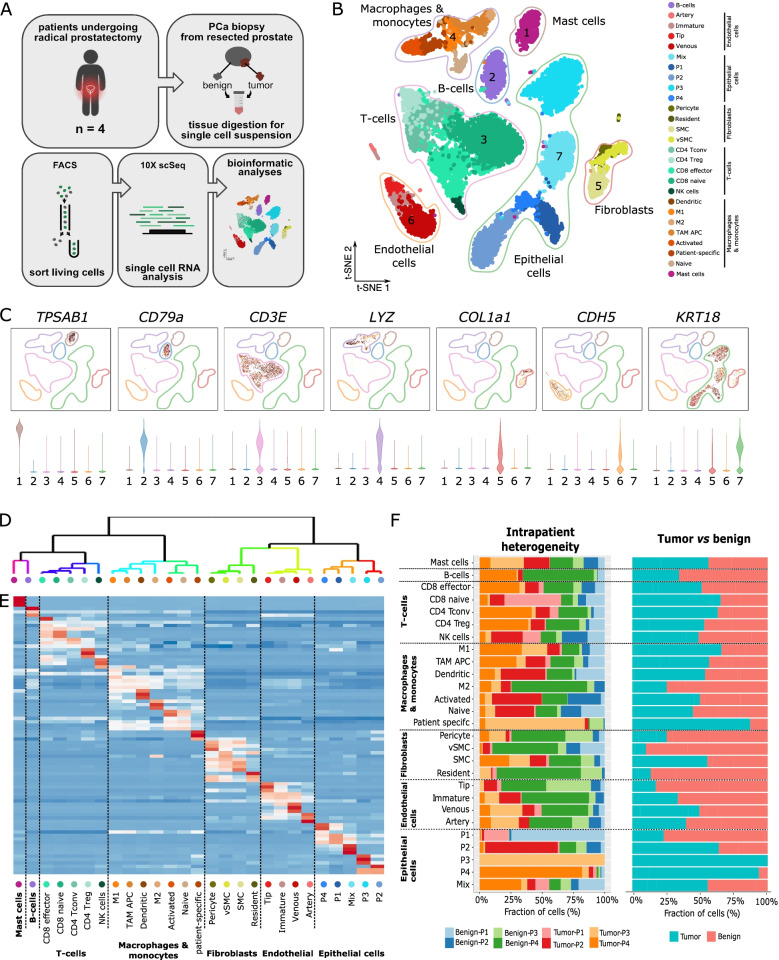
Single-cell catalogue of the prostate tumor microenvironment. **A** Graphical representation of the experimental design. **B** t-SNE analysis of 16,529 freshly-isolated PCa TME cells. **C** Top panel: t-SNE plots color-coded for the indicated marker genes. Bottom panel: violin plots quantifying the expression of the indicated gene. Note, the numbers on the x-axis correspond to the cluster numbers shown in Fig. 4B. **D** Dendrogram visualization of hierarchical clustering analysis on gene signature correlations of highly variable genes. Note, the dots on the x-axis are color-coded, as in Fig. 4B. The order is the same as in Fig. 4E. **E** Heatmap analysis of the top 5 marker genes of all subtypes in the PCa TME. **F** Left panel: horizontal bar graphs indicating the relative composition of the TME cells across patients. Right panel: horizontal bar graphs indicating the relative cell composition in tumor and benign samples

To investigate the expression of TEC markers in general and CXCL12 in particular in the full TME, we performed scRNA-seq analysis on RP tissue from four PCa patients (Fig. 4A, and Table S2). t-SNE visualization and clustering of 16,529 high-quality cells revealed B-cells, T-cells, NK-cells, macrophages and monocytes, mast cells, epithelial (and cancer) cells, fibroblasts and EC (Fig. 4B-C). Subclustering of individual cell populations and annotation using canonical marker genes revealed a total of 27 subpopulations with highly distinct gene expression signatures (Fig. 4D-E, Table S5).

**Fig. 5:**
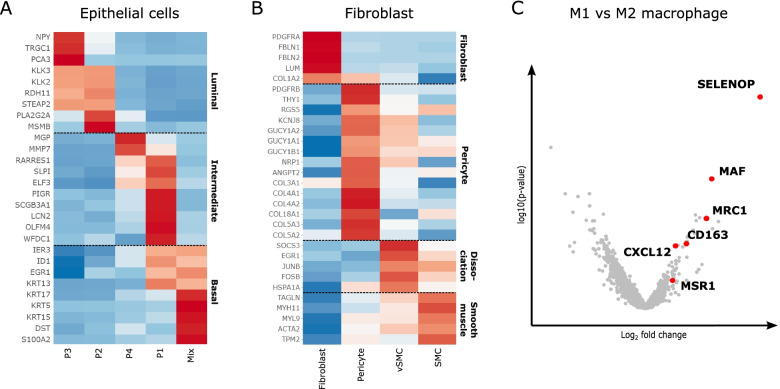
Detailed analysis of the TME stromal cell compartment. **A** Heatmap analysis of the expression of the PAM50 signature in epithelial cells. **B** Heatmap analysis of the expression of a curated list of previously published fibroblast and stromal cell markers and other highly upregulated genes. **C** Volcano plot of differential analysis of macrophages with an M1 *vs* M2 signature. Canonical M2 marker genes are indicated

Interestingly, several subpopulations were differentially distributed across patients and in tumor *versus* benign prostate tissue (Fig. 4F). For example, epithelial (and cancer) cells were highly patient-specific, whereas stromal cells were distributed more homogeneously across patients. A detailed analysis of individual subpopulations revealed four lymphocyte subcluster (CD8 naïve and effector cells, conventional CD4^+^ T-helper and regulatory T-cells) (Fig. 4E). Furthermore, we identified epithelial cells expressing gene signatures consistent with luminal, intermediate and basal subtypes of the PAM50 gene signature [31]. Two epithelial clusters highly expressed the cancer cell marker KLK3, also known as PSA, the most common marker used for PCa diagnosis and recurrence [32] (Fig. 5A and Table S5). We subclassified fibroblasts into resident fibroblasts and stromal cells (pericytes and vascular smooth muscle cells) based on recently described gene signatures (Fig. 5B and Table S5) [33]. Macrophages and monocytes were subclustered into macrophages expressing M1 and M2 signatures, tumor-associated antigen-presenting macrophages (TAM APC), dendritic cells and naïve as well as activated monocytes (Fig. 4D-E). A differential analysis of macrophages with M1 *versus* M2 signatures revealed known and novel markers of the pro-tumorigenic M2 phenotype (Fig. 5C, Table S5). Interestingly, compared to M1 macrophages, the pro-angiogenic M2 macrophages display highly upregulated CXCL12 mRNA expression (Fig. 5C, Table S5).

### Characterizing tumor endothelial cells subtypes

**Fig. 6:**
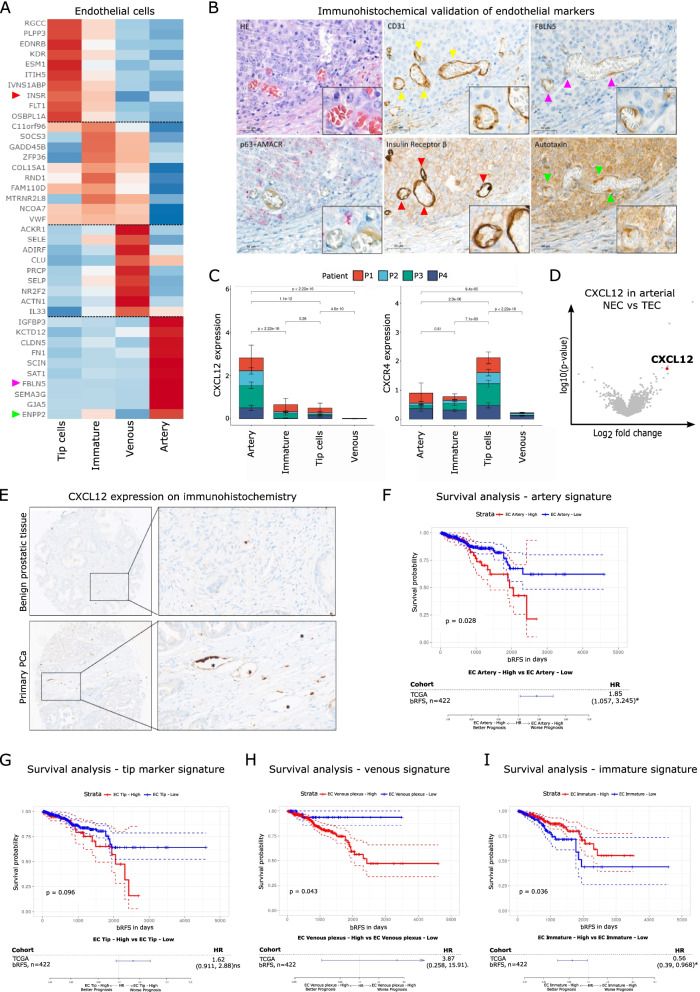
Single-cell analysis of the endothelial compartment. **A** Heatmap analysis of the top 10 marker genes of the four EC clusters. Note, the colored arrowheads on the left indicate genes validated by immunohistochemistry (INSRß, FBLN5, Autotaxin (ENPP2)). **B** Micrographs of immunohistochemistry probing the indicated genes in the tumor vasculature. The left upper panel shows the H&E staining for reference. The left lower p63/AMACR double-staining for PCa validation. **C** Representation of CXCL12 and CXCR4 expression (y-axis) for each endothelial subtype (x-axis). The statistical significance of the CXCR4/CXCL12 expression difference between the groups was tested using Wilcoxon's test. **D** Volcano plot of differential analysis of normal vs tumor arterial EC. CXCL12 is indicated in red. **E** Micrographs of immunohistochemistry probing CXCL12. Note the high expression levels of CXCL12 in the tumor vasculature. **F**- **I**. Kaplan–Meier curves and hazard ratio analysis with patients stratified based on the high or low expression of the EC artery signature (**F**), EC tip marker gene signature (**G**), EC venous signature (**H**) and EC immature signature (**I**). Information from the EC gene expression profiles was condensed into a signature summary using Gene Set Variation Analysis (GSVA). The EC marker gene signature expression cutoff was determined using the results of the GSVA and the R package “maxstat”

Our analysis showed four previously described populations of tumor EC expressing signatures of arteries, venous plexus, immature vasculature and tip cells (Fig. 6A) [11]. These populations expressed highly distinct markers, several of which were validated by IHC (Fig. 6A-B). Arteries were characterized by expression of ectonucleotide pyrophosphatase-phosphodiesterase 2 (ENPP2), gap junction protein α 5 (GJA5) and fibronectin (FN1), known markers for arterial EC differentiation regulating vascular integrity, elastic fiber assembly, vasotonus regulation and suppression of arterial calcification [34]. Top venous markers were atypical chemokine receptor-1 (ACKR1) and von Willebrand factor (vWF) expression also important factor in venous EC [35, 36]. Tip cells were characterized by expression of the endothelin receptor B (EDNRB), regulator of cell cycle gene (RGCC), vascular endothelial growth factor receptor 2 (KDR) and receptor tyrosine kinase Flt-1 (VEGFR1) genes associated with VEGF signaling, EC migration, extracellular matrix (ECM) formation and cytoskeletal/actin remodeling. The TEC marker CXCL12 that we identified through bulk RNA-seq analysis and an unbiased analysis of pro-angiogenic M2 macrophages was highest expressed in arterial EC (Fig. 6C). Differential analysis of NEC *versus* TEC showed that CXCL12 was among the highest upregulated genes in arterial TEC, compared to arterial NEC (Fig. 6D). Protein-level validation of CXCL12 using IHC was consistent with our single-cell analysis and revealed highly specific expression in the TEC of primary PCa samples (Fig. 6E). Finally, TCGA data analysis revealed that patients that highly express the arterial EC signature (defined as the top 10 specific arterial EC marker genes) have a decreased recurrence-free survival after RP (Fig. 6F). Furthermore, the EC subpopulations gene signatures of tip, venous and immature cells correlated with decreased survival (Fig. 6G-I).

In summary, the characterization of TEC subpopulations revealed that CXCL12 highly upregulated in arterial TEC and that the identified specific vasculature alterations are important prognostic markers.

### Interaction analysis highlights the CXCR4/CXCL12 axis as a candidate anti-angiogenic target

**Fig. 7:**
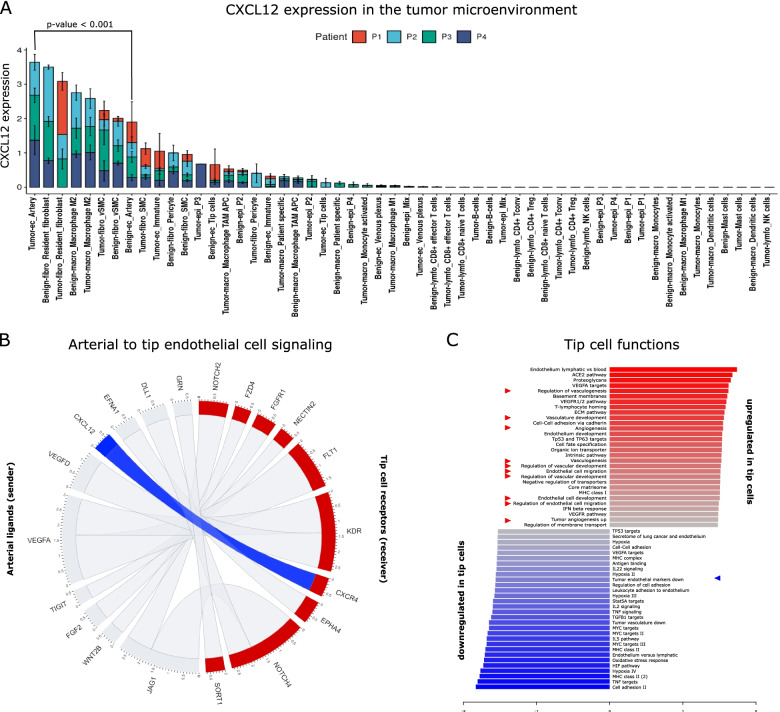
Receptor-ligand interaction analysis reveals the CXCR4/CXCL12 axis as a therapeutic target. **A**. CXCL12 expression in individual cell subtypes in the TME. The statistical significance of the CXCL12 expression difference between the Tumor—Benign Artery subtypes was tested using Wilcoxon's test. **B** Circos plot showing the results of a receptor-ligand interaction analysis using the CellPhoneDB algorithm. **C** Waterfall plot shows the top up-and downregulated pathways in tip cells compared to other endothelial cell phenotypes

Taking advantage of our profiling efforts of the entire PCa TME, we next assessed CXCL12 expression in arterial EC compared to all stromal cell compartments. Interestingly, CXCL12 is significantly higher expressed in cell (sub)types previously associated with angiogenesis (endothelial cells, fibroblasts and M2 macrophages) compared to all other cell types (Fig. 7A). Notably, tumor arterial EC expressed CXCL12 the highest, even among angiogenic phenotypes (Fig. 7A). To better understand the role of arterial EC derived CXCL12 in the PCa TME, we performed an interaction analysis using CellPhoneDB (Fig. 7B). These analyses consistently revealed paracrine signaling from CXCL12 to its cognate receptor CXCR4 on immune and, even more importantly, on tip cells (Fig. 7B). Tip cells highly overexpress CXCR4 compared to other EC phenotypes and are characterized by induced pro-angiogenic pathways associated with vascular development, vasculogenesis, EC development and tumor angiogenesis (Fig. 7C, Table S4). These findings strongly suggest that tip cells contribute to a pro-tumorigenic TME and that targeting the CXCR4/CXCL12 axis to inhibit angiogenesis might offer a potential therapeutic target.

Investigating a potential interaction of CXCR4/CXCL12 axis with the AR, the most common therapeutic target for systemic therapies, IHC revealed AR expression in EC more affecting tumor areas compared to stromal tissue (Fig. S4). ScRNA-seq data show AR expression mainly in venous and immature EC, thus we assume the interaction of the endothelial AR and the CXCR4/CXCL12 axis should be further explored (Fig. S5).

### In vitro and in vivo functional validation of CXCR4/CXCL12 inhibition in PCa

**Fig. 8:**
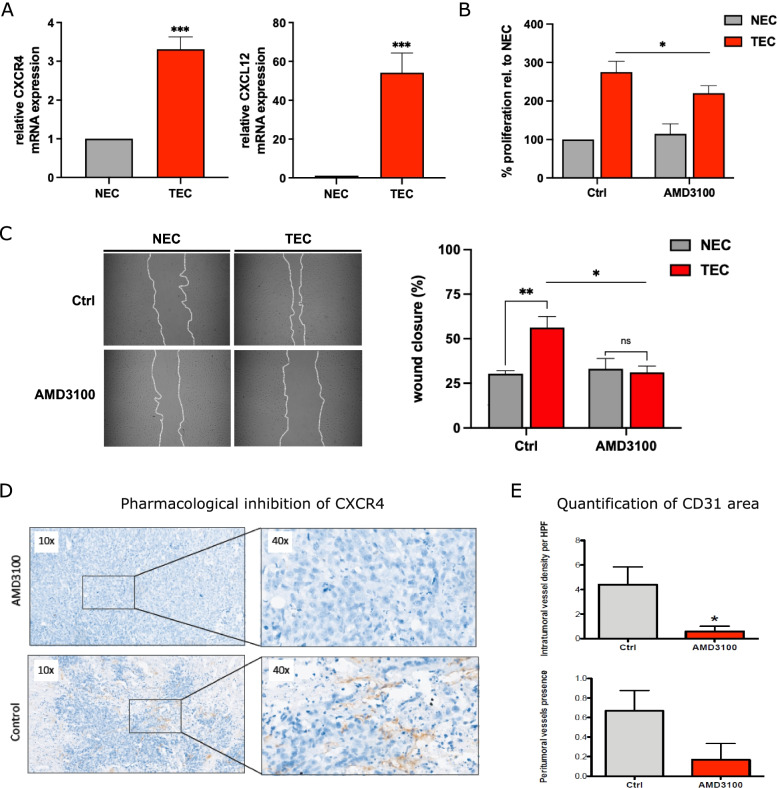
In vitro and in vivo validation of targeting the CXCR4/CXCL12 axis to inhibit angiogenesis. **A** Quantitative RT-PCR analyses of CXCR4 and CXCL12 mRNA expression in prostate NEC and TEC (mean +—SEM; *n* = 3, *p* < 0.001). **B** EZ4U cell prolifaration assay with NEC and TEC treated with 10 µM AMD3100 for 24 h (mean +—SEM; *n* = 3, *p* < 0.05). **C** Micrographs (left) and quantification of wound closure (right) of NEC and TEC migration in scratch wound assay. Cells were treated with 10 µM AMD3100 for 24 h (mean +—SEM; *n* = 3, *p < 0.05, **p < 0.01). **D** Micrographs showing immunohistochemistry results after probing for the endothelial marker CD31 in tissues treated, or not, with the CXCR4 inhibitor AMD3100. **E** Quantification of intratumoral and peritumoral microvessel density in control vs AMD3100 treated murine PCa tissue

By using RT-PCR analysis, we could demonstrate that the expression of CXCL12 and its cognate receptor CXCR4 are upregulated in TEC compared to NEC (Fig. 8A). Of note, the CXCL12/CXCR4 axis is known to promote tube formation, which was confirmed by repression of neovascularization by HUVEC by the CXCR4 antagonist AMD3100 [37]. In concordance with the concept that CXCR4/CXCL12 signaling triggers neovascularization in PCa TEC, we confirm that the CXCR4 antagonist AMD3100 efficiently reduces of the proliferative capacity of TEC (Fig. 8B) and efficiently represses elevated cell migration of TEC (Fig. 8C). Importantly, the impact of CXCR4 modulation by AM3100 treatment was exclusively visible in TEC, whereas NEC migration or proliferation were not affected.

Taking advantage of a previous murine study that showed a synergistic effect of chemotherapy and blockade of the CXCL12 receptor CXCR4 in PCa, we performed IHC of the vasculature in this model [23]. All tumors of control mice (*n* = 5) exhibited CD31^+^ vessels with three tumors showing numerous, distributed vessels and two tumors showing focal hypervascularization. Four out of 5 tumors harbored intratumoral vessels. Out of 6 tumors of mice treated with the CXCR4 antagonist AMD3100, 2 showed focal intra- or peritumoral vessels while there was no tumor with distributed intratumoral vessels as seen in control mice (Fig. 8D-E). AMD3100 did not affect tumor shrinkage as monotherapy; however, after combinational therapy with docetaxel, a synergistic effect was seen, and significant tumor shrinkage could be observed.

## Discussion

The TME orchestrates cancer progression and modification of TME components has proven clinical efficacy in several cancers like renal cell cancer or non-small cell lung cancer (NSCLC) [1, 3, 38]. However, in PCa, the most common cancer in men, TME modifying therapies like anti-angiogenic drugs or immune-checkpoint inhibitors did not show relevant clinical activity yet [39, 40].

Therefore, characterization of the vascular network influencing TME composition as well as PCa progression and metastasis formation is of unmet need and a milestone for further TME targeting in PCa. We here provide the first comprehensive characterization of the prostate TME focusing on the EC compartment. Key findings of our study are: i) PCa TEC differ in cytological, junctional and functional properties from NEC; ii) TME mapping of treatment naïve PCa by scRNA-seq identified 27 subpopulations with distinct gene expression signatures; iii) novel PCa specific TEC markers were identified and orthogonally validated; iiii) we identified a prostate TEC specific artery EC gene signature associated with decreased survival; iv) CXCR4/CXCL12 axis is as novel promising anti-angiogenic target which was validated in vitro and in vivo.

In depth mapping of TEC and NEC phenotypes generated from cultured and freshly isolated PCa patient tissue unveiled that TEC are imprinted by the PCa TME and retain a rewired functional phenotype even when ex vivo expanded over-time. In line with other tumors, TEC differ morphologically and functionally from their normal physiologic counterparts (NEC). However, the hyperactive status seems to be not as pronounced as in highly angiogenic tumor entities like NSCLC [11].

Analysis of bulk RNA-seq data from cultivated prostate TEC and NEC revealed an upregulation of pathways linked to TME hallmarks of cancer, such as tumor vasculature and collagen modification. Remarkably, also NEC acquire in culture certain “TEC-like” features as VEGF and hypoxia signaling, which might be explained by the artificial ex vivo growth conditions. This TEC conditioning has also been reported by other publications [7, 11].

Next, collagen hydroxylation has been identified in NSCLC as a potential novel TEC targeting strategy [9]. Of note, most upregulated genes are involved in these pathways as PTGIR and PRDM8 regulating vasodilatation, platelet aggregation or vascular smooth muscle cell proliferation. Our analysis revealed the CXCR4/CXCL12 axis in the TME/TEC interaction and the arterial EC to tip TEC communication. In general, cytokines and chemokines are mediators of PCa progression [41] and CXCL12 gradients are essential for tumor cell transmigration and metastasis [23, 28]. However, the source of CXCL12 production in the TME, its expression distribution profile, and the exact mechanism of action remained elusive so far.

We here identified CXCL12 as a novel prostate specific TEC candidate. Clinical relevance of our finding is supported by inferior recurrence-free survival in PCa patients with high CXCL12 expression. This complements recent data demonstrating a link between increased PDGFR/CXCL12 expression to decreased survival of patients with PCa [28]. Interestingly, scRNA-seq revealed that CXCL12 is highest expressed in artery TEC as well as (although to a lower extent) in immature EC and tip cells, fibroblasts and macrophages. Putting this in perspective with the results from bulk RNA-seq EC culturing might promote arterial or tip cell outgrowth contributing to high CXCL12 expression levels. CXCR4/CXCL12 may also modulate PCa cell migration and invasion [42], however in our study receptor-ligand interaction analyses predicted strong communication between arterial TEC-derived CXCL12 with its cognate receptor CXCR4 on angiogenic tip EC. Our descriptive data are supported by in vitro expriments demonstrating that CXCL12 and CXCR4 are significantly upregulated in TEC compared to NEC. In addition, the CXCR4 antagonist AMD3100 (plerixafor®) efficiently represses elevated cell proliferation and migration of TEC but not of NEC. Additionally, using a subcutaneous xenograft mouse model, we show for the first time that the CXCR4 inhibitor AMD3100 has anti-angiogenic solid properties by decreasing vessel number and density. Therefore, the interaction of CXCR4/CXCL12 represents a novel and promising vasculature targeting therapeutic option by the reducing novel blood vessel formation. In addition, a significantly higher tumor shrinkage was observed when AMD3100 was combined with standard therapies than chemotherapy or 2nd generation androgen deprivation agents like enzalutamide [23, 43]. Based on these findings, we suggest testing the CXCR4/CXCL12 axis in combination with standard treatment options in metastatic castration-resistant PCa.

In addition to identification and validation of PCa TEC, out study deeply mapped the PCa TME. Similarly, another scRNA-seq study described a similar PCa TME composition including 36,424 cells from 13 patients [44]. The strength of our study is the homogenous systemic treatment naïve patient collective as well as the strict selection criteria (e.g. only intermediate or high-risk PCa without systemic tumor spread confirmed in the preoperative staging before RP). Finally, our study provides a publicly available resource for data exploration using the Unicle webtool (https://unicle.life/portals/).

The scRNA-seq data also allow in-depth analysis and understanding of the various and multifaceted PCa subpopulations. As an example, the tumor endothelium can be classified into four distinct subclusters also enabling the annotation of INSR as PCa tip cell marker and Fibulin5 and ENPP2 as PCa artery specific markers. Of note, we confirmed our gene expression findings in PCa patients using IHC. In addition to our prime PCa TEC target CXCR4/CXCL12, we propose also FBLN as novel artery targeting strategy PCa target. In line with this hypothesis, FBLN5 inhibition exerts strong anti-angiogenic effects by reducing endothelial cell viability and interfering with the signaling pathways of the Ang-1/TIE-2 receptor axis [45]. ENPP2 (Autotaxin) interacts with EC permeability, and ENPP2 expression is associated with acquired resistance against the anti-angiogenic agent sunitinib in renal cell cancer [46]. Generally, INSR is not a deliberate target of cancer therapies, as the implementation of INSR blocking therapies appears particularly problematic given the crucial role of INSR in glucose metabolism. Rather, the attention focused on the homologous insulin-like growth factor receptor 1 (IGF1R) however, with disappointing results from clinical studies [47]. Indeed, the present survey indicates the INSR is highly expressed on tip EC. Thus, one can consider the inhibition or reversion of artery signature by a drug specifically targeting the INSR on tip EC, possibly overcoming or at least reducing the glucose-based side effects.

Our here identified arterial EC signature, of ten artery specific EC markers, prognosticates inferior biochemical recurrence-free survival, again underpinning the biological importance of TEC for the biological behavior of PCa. Of note also other EC signatures (immature, venous and tip) pose prognostic relevance and based on these findings anti-angiogenic approaches in PCa, in particular the combination of CXCR4 inhibitors with immunotherapeutic approaches, AR modulation should be reconsidered despite the historic disappointments with anti-VEGF approaches. In addition, the AR might be an interesting target in PCa TEC and should be further explored.

We acknowledge the limitations of our work. First, the inferred biological role for each EC phenotype requires functional validation. Second, scRNA-seq studies with higher patient and cell numbers are required to exclude inter- and intra-patient tumor heterogeneity, which could impact that TEC from different regions may behave differently. Our exploratory work provides the basis for future studies that could highlight this important aspect and further work out the role of exclude inter- and intra-patient tumor heterogeneity. In addition, whole-exome sequencing, spatial resolution of these patients would be essential to obtain information about genetic variations within the PCa cells. Moreover, we speculate that therapy pressure (e.g. hormonal therapy) might significantly affect the TME, which may be of clinical relevance in terms of novel therapeutic targets and biomarkers.

## Conclusion

We isolated and comprehensively characterized TEC from human PCa using bulk and scRNA-seq and identified prognostic TEC signatures as well as potential novel therapeutic TEC targets.

## Supplementary Information

**Additional file 1: Supplementary figure 1.** Workflow for tumor and benign prostate tissue analysis.

**Additional file 2:** **Supplementary figure 2. **Overview of sample collection for bulk RNA-seq and functional analyses.

**Additional file 3:** **Supplementary figure 3. **A) Box and whiskers plots of CXCL12 and CXCR4 expression among the three Gleason score ranks (≤6, 7, ≥8). Testing whether samples originate from the same distribution was performed using a non-parametric Kruskal-Wallis’ test, while the identification where a stochastic dominance occurs was concluded using pairwise non-parametric Wilcoxon’s tests. B) Box and whiskers plots of CXCL12 and CXCR4 expression between prostate adenocarcinoma acinar and other types. Pairwise comparisons are performed using non-parametric Wilcoxon’s tests. C) Box plots of PSA levels between CXCL12 and CXCR4 high-low expression ranks. The expression level cutoff was defined using the R package “maxstat”. Pairwise comparisons are performed using non-parametric Wilcoxon’s tests. D) Box and whiskers plots of CXCL12 and CXCR4 expression between PSA high-low level ranks. The PSA levels were defined using an empirical cutoff threshold of 10 ng/ml. Pairwise comparisons are performed using non-parametric Wilcoxon’s tests. E) Correlation between PSA levels and CXCR4/CXCL12 expression. The correlation analysis was performed using Pearson’s correlation tests at a significance level (α) of 0.05.

**Additional file 4:** **Supplementary figure 4. **A) IHC on consecutive slides for CD31 (left side) and AR (right side) in stromal tissue with strong positive expression for CD31 and weak expression for AR in endothelial cells. B) IHC on consecutive slides for CD31 (left side) and AR (right side) in cancer tissue with strong positive expression for CD31 and positive expression for AR in endothelial cells and strong expression of AR in cancer cells.

**Additional file 5:** **Supplementary figure 5. **Bar plot of androgen receptor expression (y-axis) across EC phenotypes (x-axis). The difference between the means was tests using non-parametric Wilcoxon’s tests.

**Additional file 6:** **S1. **Key resources.

**Additional file 7:** **S2.** Patient characteristics.

**Additional file 8:** **S3**. Bulk RNA-seq: differentially expressed genes between cultured TEC and NEC.

**Additional file 9:** **S4**. Pathway analysis of bulk and single-cell RNA sequencing: list of pathways differentiating NEC vs TEC as inferred from the GSEA analysis. scRNA-seq_tip cell_pathways: Tip cell-enriched pathways as inferred from the GSEA analysis.

**Additional file 10:** **S5. **Single-cell RNA sequencing: *scRNAseq_DGE:* differentially expressed genes between tumor and benign conditions, *scRNAseq_DGE_arterial_NEC_vs_TEC:*gene list discriminating NEC *versus* TEC by scRNAseq,*All_Clusters_GeneList:*maker genes differentiating the cell clusters by scRNAseq, *M1_vs_M2_GeneList:* gene list differentiating between M1 and M2 macrophages by scRNAseq, *Endothelial_Clusters_GeneList:*gene list for Tip cells, Immature cells Venous plexus and Artery Endothelial clusters by scRNA-seq.

## Data Availability

The accession number for all raw sequencing data are available in ArrayExpress / GEO under accession numbers: GSE193337 (secure token: srgpkcwkvvejfgt). To ensure data accessibility to non-bioinformaticians, reproducibility, and resource value, we made our scRNA-seq data available for further exploration via an interactive webtool: https://unicle.life/portals/ Using this tool users can interactively visualize gene expression and clustering on t-SNE, search marker genes for all subclusters, and export gene expression data. Most important materials and products are listed in supplementary Table S1.

## Abbreviations

AR: Androgen receptor
EC: Endothelial cells
ECM: Endothelial cell growth medium
IF: Immunoflourescence
IHC: Immunohistochemistry
INSR: Insulin receptor
NEC: Normal endothelial cells
NSCLC: Non-small cell lung cancer
PSA: Prostate specific antigen
PCa: Prostate cancer
PCA: Principal component analysis
RP: Radical prostatectomy
scRNA-seq: Single cell RNA sequencing
RNA-seq: Bulk RNA sequencing
TMA: Tissue microarray
TME: Tumor microenvironment
TEC: Tumor endothelial cells
