# Supplementary material for: Comprehensive characterization of the prostate tumor microenvironment identifies CXCR4/CXCL12 crosstalk as a novel antiangiogenic therapeutic target in prostate cancer
Source: Mol Cancer. 2022 Jun 18;21:132. doi: 10.1186/s12943-022-01597-7 (PMC9206324; doi:10.1186/s12943-022-01597-7)
Supplement: Supplementary file 1 — Additional file 1: Supplementary figure 1. Workflow for tumor and benign prostate tissue analysis. [file 12943_2022_1597_MOESM1_ESM.pdf]

Central slice of radical prostatectomy

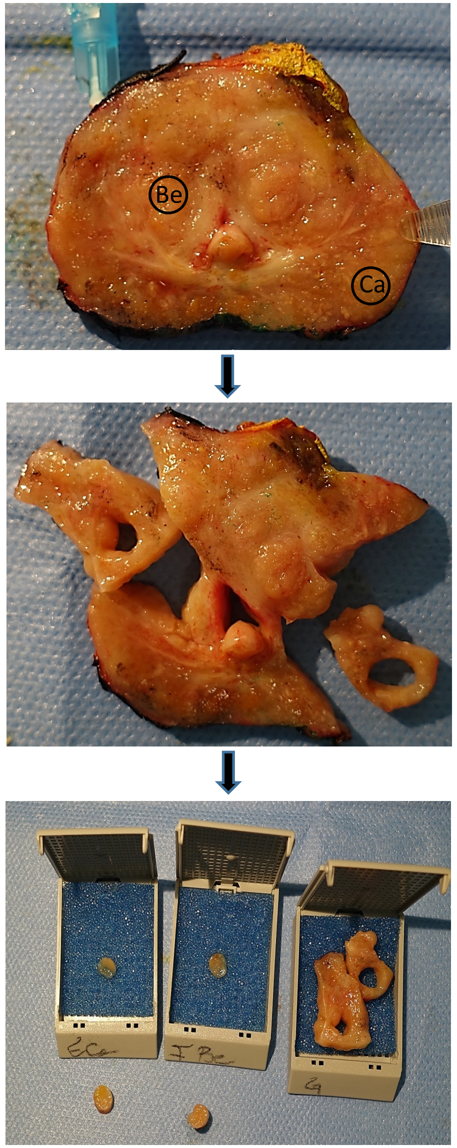

Paired benign (TZ left) and cancer (PZ right) punch biopsies plus surrounding tissue and biopsy tops for validation

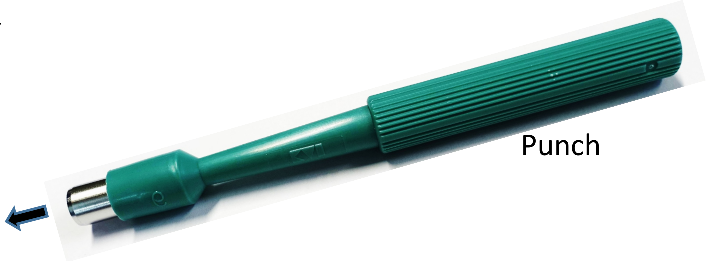

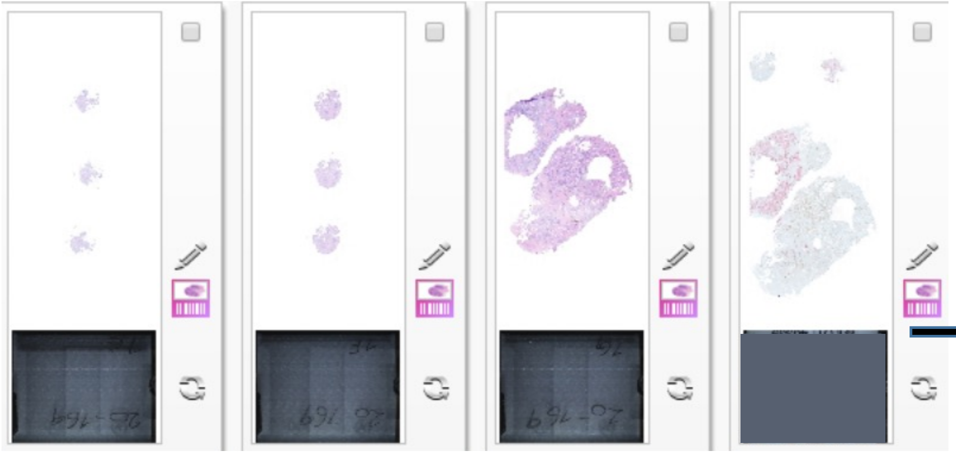

HE histological and IHC double staining (p63/AMACR) validation of biopsy top and surrounding tissue

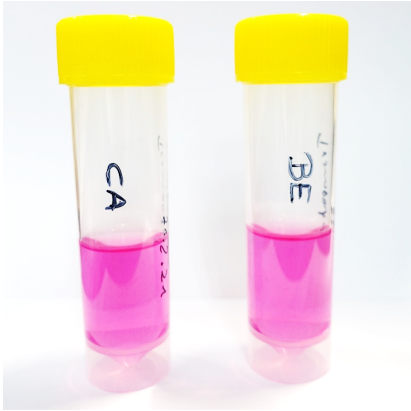

Biopsies in transport medium for tissue digestion for NEC/TEC cultures or scRNA-seq

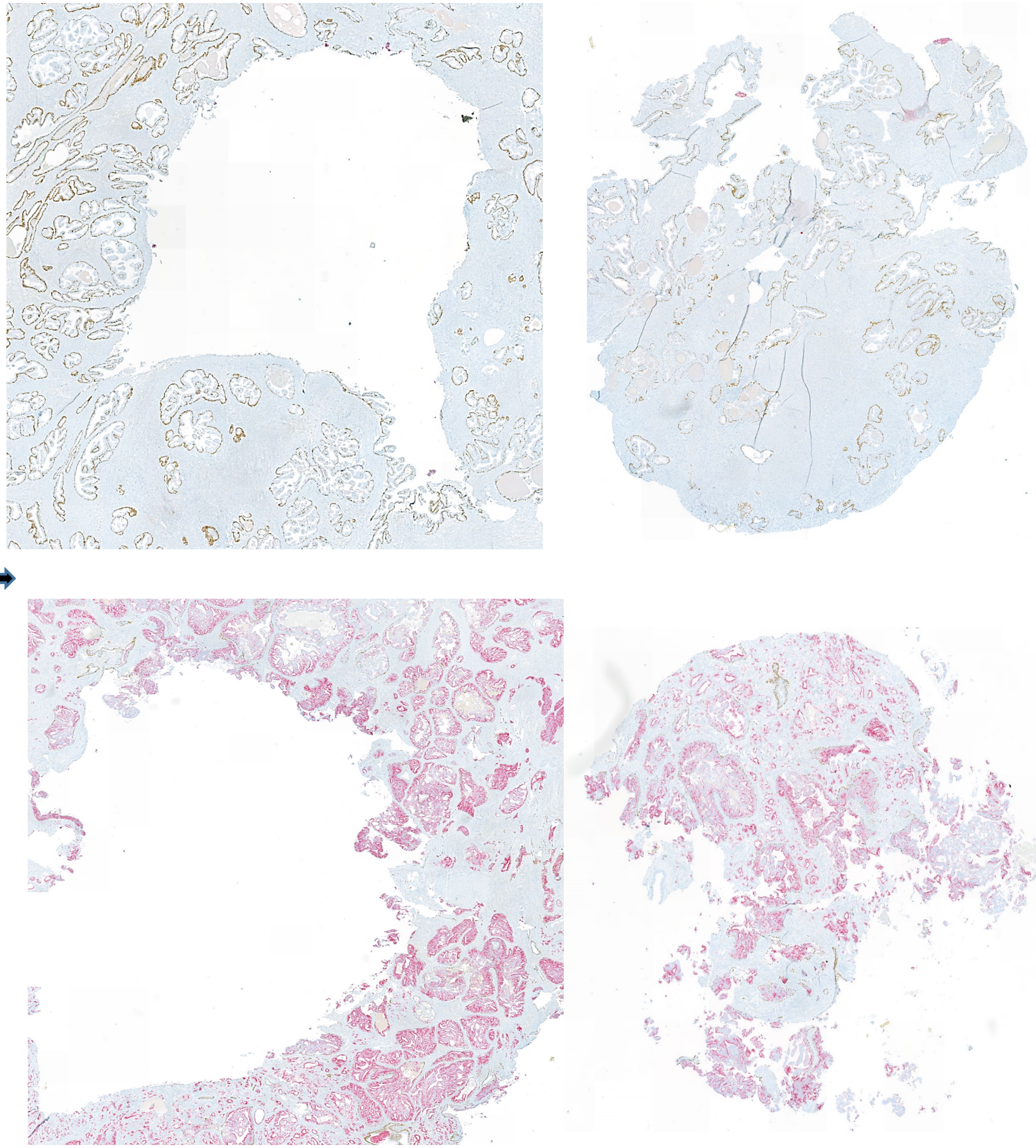

20x magnification of IHC double staining (p63/AMACR) of biopsy top and surrounding tissue
(upper - Benign: AMACR-/p63+, lower - Cancer: AMACR+/p63-)
