# Supplementary figures and images for: Comprehensive characterization of the prostate tumor microenvironment identifies CXCR4/CXCL12 crosstalk as a novel antiangiogenic therapeutic target in prostate cancer
Source: Mol Cancer. 2022 Jun 18;21:132. doi: 10.1186/s12943-022-01597-7 (PMC9206324; doi:10.1186/s12943-022-01597-7)

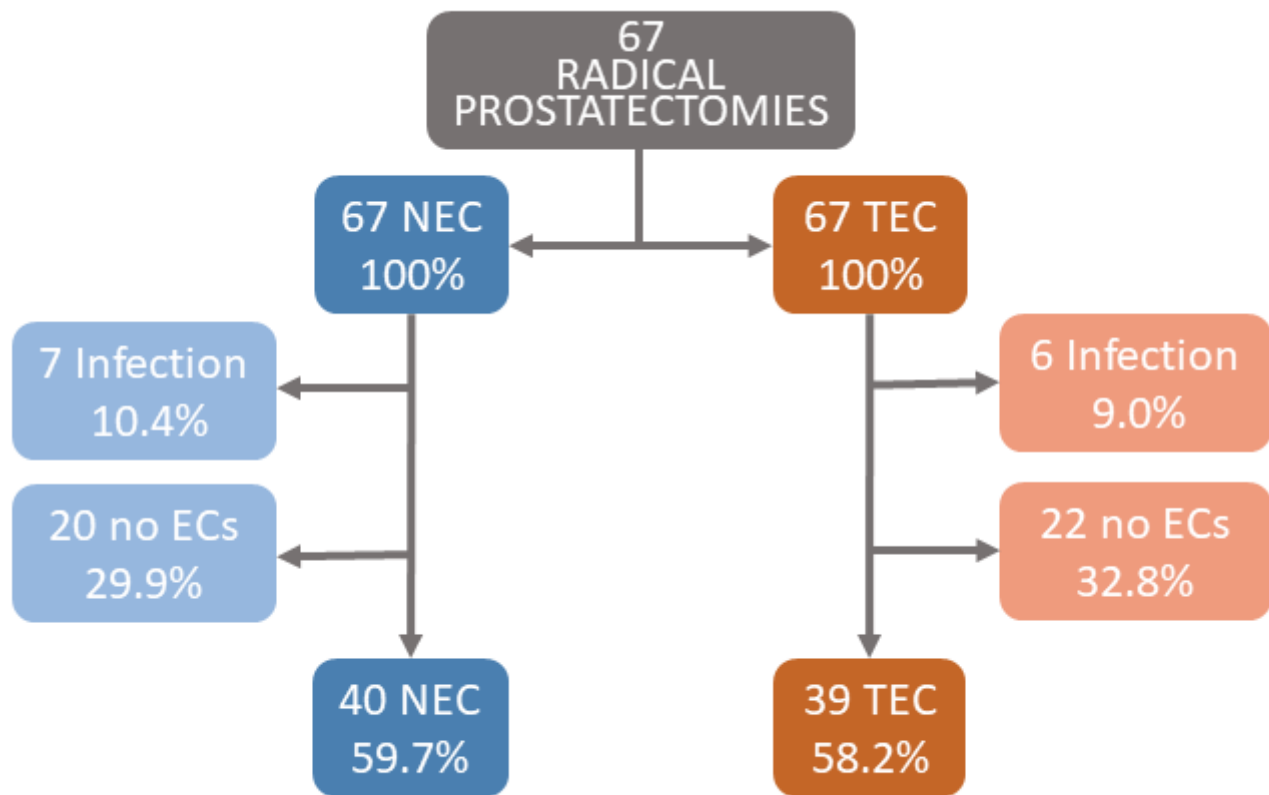

Supplement: Supplementary file 2 — Additional file 2: Supplementary figure 2. Overview of sample collection for bulk RNA-seq and functional analyses. [file 12943_2022_1597_MOESM2_ESM.pdf]

*CD 31*

*Androgen receptor*

**A**

Stromal tissue

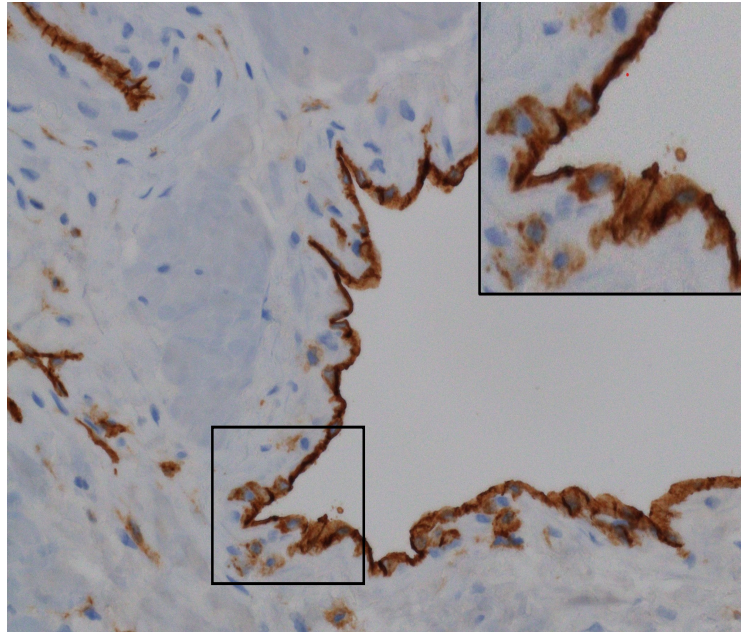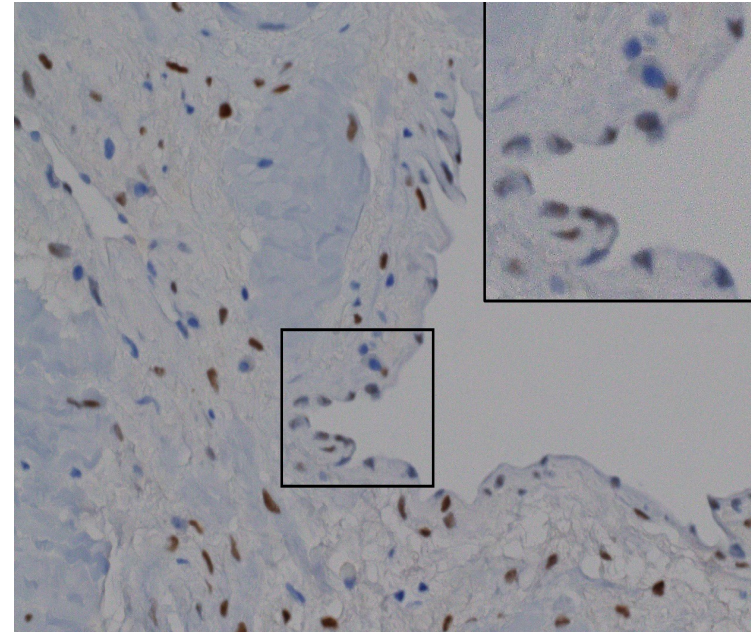

**B**

Cancer tissue

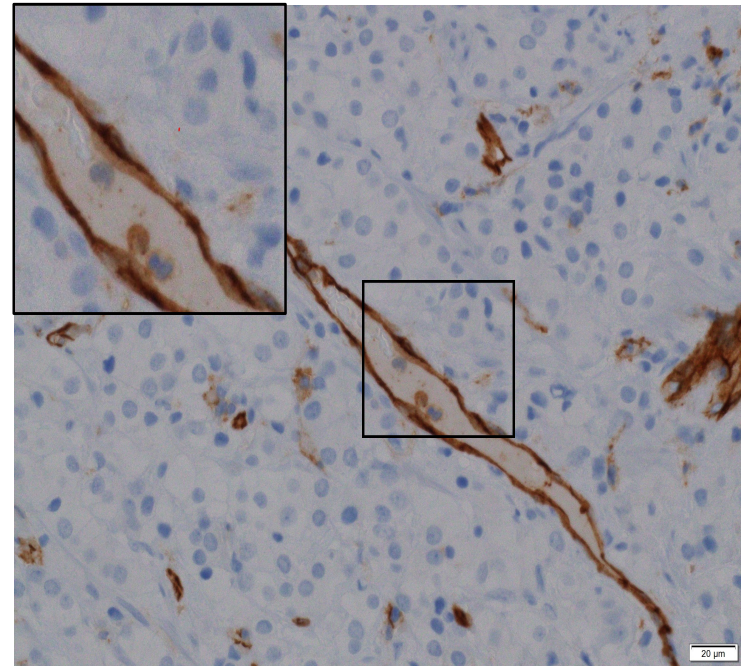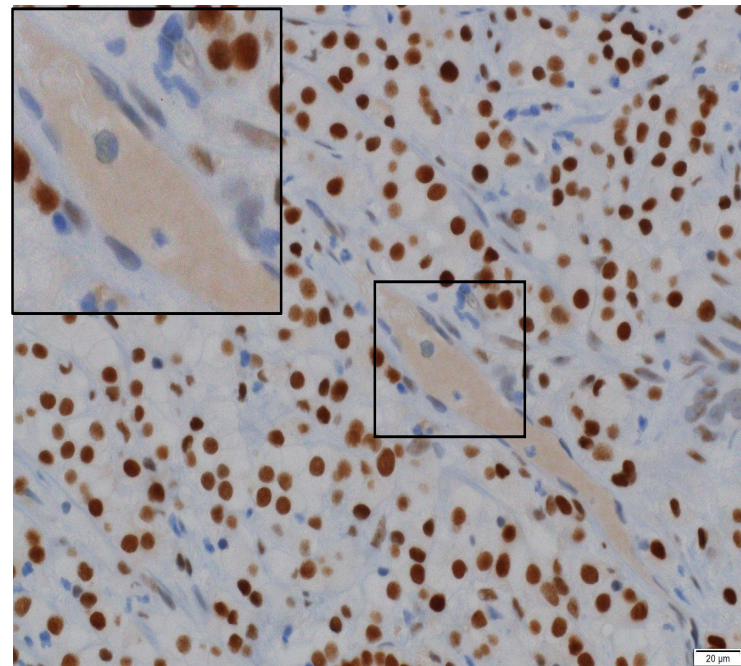

Supplement: Supplementary file 4 — Additional file 4: Supplementary figure 4. A) IHC on consecutive slides for CD31 (left side) and AR (right side) in stromal tissue with strong positive expression for CD31 and weak expression for AR in endothelial cells. B) IHC on consecutive slides for CD31 (left side) and AR (right side) in cancer tissue with strong positive expression for CD31 and positive expression for AR in endothelial cells and strong expression of AR in cancer cells. [file 12943_2022_1597_MOESM4_ESM.pdf]

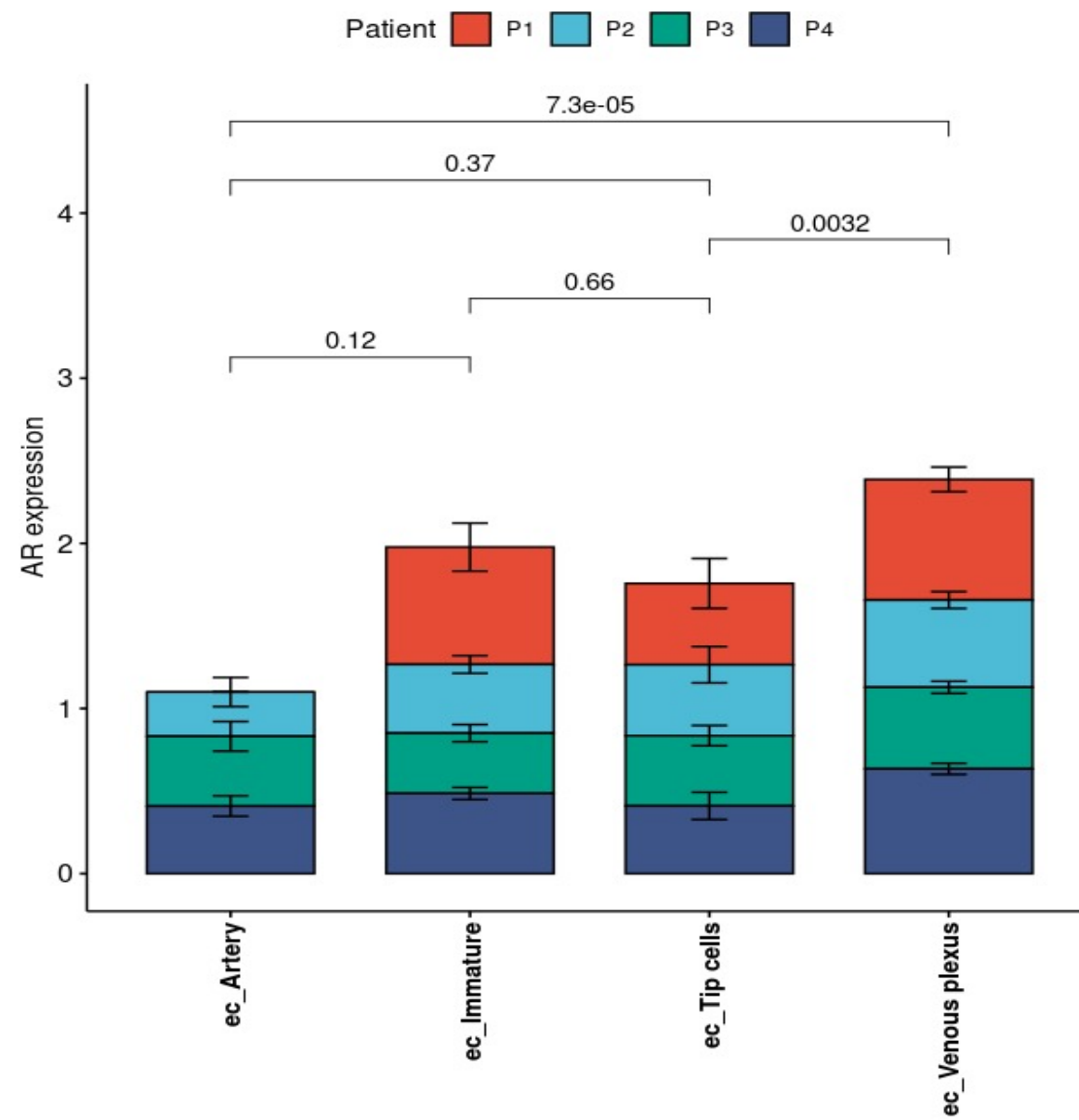

Supplement: Supplementary file 5 — Additional file 5: Supplementary figure 5. Bar plot of androgen receptor expression (y-axis) across EC phenotypes (x-axis). The difference between the means was tests using non-parametric Wilcoxon’s tests. [file 12943_2022_1597_MOESM5_ESM.pdf]
