# Supplementary material for: Comprehensive characterization of the prostate tumor microenvironment identifies CXCR4/CXCL12 crosstalk as a novel antiangiogenic therapeutic target in prostate cancer
Source: Mol Cancer. 2022 Jun 18;21:132. doi: 10.1186/s12943-022-01597-7 (PMC9206324; doi:10.1186/s12943-022-01597-7)
Supplement: Supplementary file 3 — Additional file 3: Supplementary figure 3. A) Box and whiskers plots of CXCL12 and CXCR4 expression among the three Gleason score ranks (≤6, 7, ≥8). Testing whether samples originate from the same distribution was performed using a non-parametric Kruskal-Wallis’ test, while the identification where a stochastic dominance occurs was concluded using pairwise non-parametric Wilcoxon’s tests. B) Box and whiskers plots of CXCL12 and CXCR4 expression between prostate adenocarcinoma acinar and other types. Pairwise comparisons are performed using non-parametric Wilcoxon’s tests. C) Box plots of PSA levels between CXCL12 and CXCR4 high-low expression ranks. The expression level cutoff was defined using the R package “maxstat”. Pairwise comparisons are performed using non-parametric Wilcoxon’s tests. D) Box and whiskers plots of CXCL12 and CXCR4 expression between PSA high-low level ranks. The PSA levels were defined using an empirical cutoff threshold of 10 ng/ml. Pairwise comparisons are performed using non-parametric Wilcoxon’s tests. E) Correlation between PSA levels and CXCR4/CXCL12 expression. The correlation analysis was performed using Pearson’s correlation tests at a significance level (α) of 0.05. [file 12943_2022_1597_MOESM3_ESM.pdf]

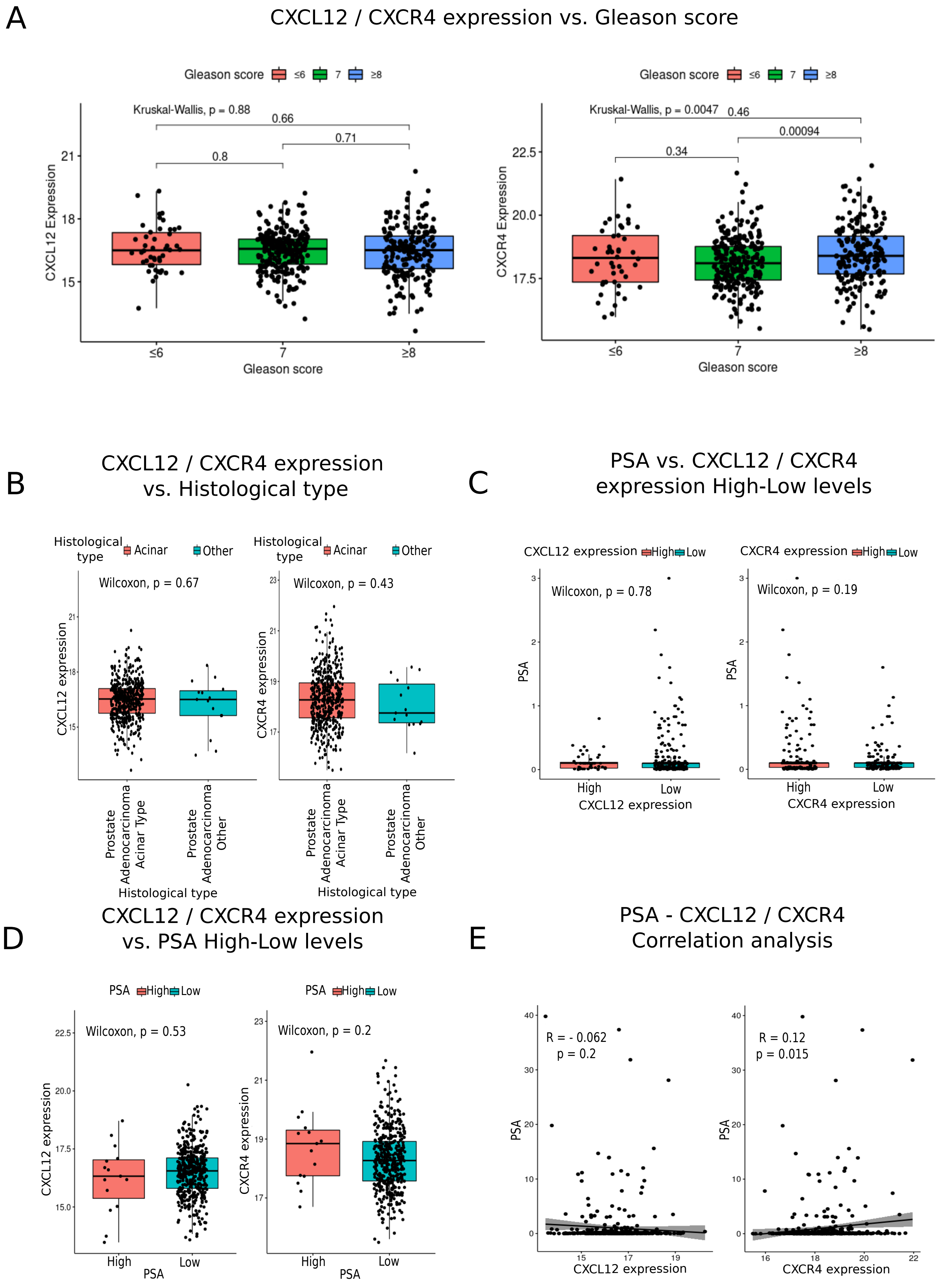

A
CXCL12 / CXCR4 expression vs. Gleason score
Gleason score ≤6 7 ≥8
Kruskal-Wallis, p = 0.88
0.66
0.71
0.8
CXCL12 Expression
21
18
15
≤6
7
≥8
Gleason score
Gleason score ≤6 7 ≥8
Kruskal-Wallis, p = 0.0047
0.46
0.00094
0.34
CXCR4 Expression
22.5
20.0
17.5
≤6
7
≥8
Gleason score
B
CXCL12 / CXCR4 expression vs. Histological type
Histological type Acinar Other
Wilcoxon, p = 0.67
CXCL12 expression
Prostate Adenocarcinoma Acinar Type
Prostate Adenocarcinoma Other
Histological type
Histological type Acinar Other
Wilcoxon, p = 0.43
CXCR4 expression
Prostate Adenocarcinoma Acinar Type
Prostate Adenocarcinoma Other
Histological type
C
PSA vs. CXCL12 / CXCR4 expression High-Low levels
CXCL12 expression High Low
Wilcoxon, p = 0.78
PSA
High
Low
CXCL12 expression
CXCR4 expression High Low
Wilcoxon, p = 0.19
PSA
High
Low
CXCR4 expression
D
CXCL12 / CXCR4 expression vs. PSA High-Low levels
PSA High Low
Wilcoxon, p = 0.53
CXCL12 expression
22.5
20.0
17.5
15.0
High
Low
PSA
PSA High Low
Wilcoxon, p = 0.2
CXCR4 expression
23
21
19
17
High
Low
PSA
E
PSA - CXCL12 / CXCR4 Correlation analysis
R = - 0.062
p = 0.2
PSA
CXCL12 expression
R = 0.12
p = 0.015
PSA
CXCR4 expression
